# Different Routes of Protein Folding Contribute to Improved Protein Production in Saccharomyces cerevisiae

**DOI:** 10.1128/mBio.02743-20

**Published:** 2020-11-10

**Authors:** Qi Qi, Feiran Li, Rosemary Yu, Martin K. M. Engqvist, Verena Siewers, Johannes Fuchs, Jens Nielsen

**Affiliations:** a Department of Biology and Biological Engineering, Chalmers University of Technology, Gothenburg, Sweden; b Novo Nordisk Foundation Center for Biosustainability, Chalmers University of Technology, Gothenburg, Sweden; c Proteomics Core Facility, Sahlgrenska Academy, Gothenburg University, Gothenburg, Sweden; d Novo Nordisk Foundation Center for Biosustainability, Technical University of Denmark, Lyngby, Denmark; e BioInnovation Institute, Copenhagen N, Denmark; Leibniz Institute for Natural Product Research and Infection Biology—Hans Knoell Institute Jena

**Keywords:** protein secretory pathway, protein folding precision, multi-omics analysis, protein production, constraint-based modeling

## Abstract

Protein folding is often considered the flux controlling process in protein synthesis and secretion. Here, two previously isolated Saccharomyces cerevisiae strains with increased α-amylase productivity were analyzed in chemostat cultures at different dilution rates using multi-omics data. Based on the analysis, we identified different routes of the protein folding pathway to improve protein production. In the first strain, the increased abundance of proteins working on the folding process, coordinated with upregulated glycogen metabolism and trehalose metabolism, helped increase α-amylase productivity 1.95-fold compared to the level in the original strain in chemostat culture at a dilution rate of 0.2/h. The second strain further strengthened the folding precision to improve protein production. More precise folding helps the cell improve protein production efficiency and reduce the expenditure of energy on the handling of misfolded proteins. As calculated using an enzyme-constrained genome-scale metabolic model, the second strain had an increased productivity of 2.36-fold with lower energy expenditure than that of the original under the same condition. Further study revealed that the regulation of *N*-glycans played an important role in the folding precision control and that overexpression of the glucosidase Cwh41p can significantly improve protein production, especially for the strains with improved folding capacity but lower folding precision. Our findings elucidated in detail the mechanisms in two strains having improved protein productivity and thereby provided novel insights for industrial recombinant protein production as well as demonstrating how multi-omics analysis can be used for identification of novel strain-engineering targets.

## Abstract (precis)

Protein folding plays an important role in protein maturation and secretion. In recombinant protein production, many studies have focused on the folding pathway to improve productivity. Here, we identified two different routes for improving protein production by yeast. We found that improving folding precision is a better strategy. Dysfunction of this process is also associated with several aberrant protein-associated human diseases. Here, our findings about the role of glucosidase Cwh41p in the precision control system and the characterization of the strain with a more precise folding process could contribute to the development of novel therapeutic strategies.

## INTRODUCTION

The protein secretory machinery in eukaryal cells is a complex network involving processes such as peptide translocation, glycosylation, protein folding, endoplasmic reticulum (ER)-associated degradation (ERAD), trafficking between the ER and Golgi apparatus, and Golgi processing and sorting (1). In yeast, this machinery is responsible for the modification and maturation of more than 550 proteins (2) which are vital to maintain cell function. High-fidelity protein folding is a crucial step in the secretory pathway (1). Misfolded proteins, which can be caused by protein overproduction, externally applied stresses such as heat or oxidative stress, or genetic factors such as genetic mutations, transcription, or translation errors, are handled by the quality control system to be either refolded or delivered to the proteasome for degradation through ERAD (3). Without proper handling by this quality control system, accumulation of misfolded proteins can be toxic to the cell. In humans, such accumulation can cause a number of severe diseases, such as Alzheimer’s disease, Parkinson’s disease, and type II diabetes (4). As a model eukaryal organism, the baker’s yeast Saccharomyces cerevisiae is a good model to study the secretory pathway and unravel the mechanisms related to these diseases (5). In addition, S. cerevisiae is also widely used as a cell factory to produce recombinant proteins (6), including biopharmaceuticals and industrial enzymes (7, 8). However, due to the complexity of the protein secretory machinery and lack of a complete understanding of its underlying mechanisms, the utility of rational strain engineering for the improvement of recombinant protein production has been limited. Correspondingly, several random approaches and semirational approaches have been performed (7), such as random mutagenesis coupled with high-throughput screening (9), omics analysis-based strain engineering (10, 11), and mathematical model-based strain engineering (12).

**FIG 1:**
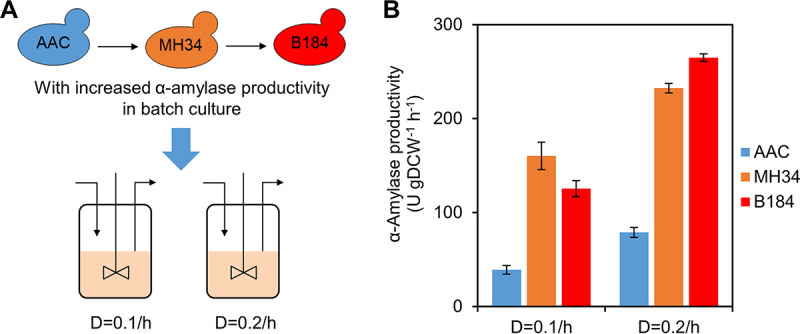
α-Amylase production of yeast strains in chemostat cultures. (A) Relationships among the strains used in this study. All three strains were grown in chemostat cultures operated at dilution rates (D) of 0.1/h and 0.2/h. (B) α-Amylase productivity of strains in steady-state chemostat cultures. Data shown are mean values ± standard errors of the means of biological duplicates. DCW, dry cell weight.

In previous studies (9, 13), we constructed the S. cerevisiae strain AAC, in which α-amylase is expressed through a *POT1* plasmid system, and used it as the original strain for several rounds of UV radiation-based mutagenesis coupled with microfluidic screening to isolate evolved strains with improved α-amylase production. Transcriptional analysis of seven evolved strains cultured in batch fermentation revealed several general rules for efficient protein production, such as an increased fermentation/respiration ratio and tuning of amino acid biosynthesis and thiamine biosynthesis (14). However, transcriptomic differences do not necessarily translate into proteomic differences, which better reflect the state of the protein secretory machinery in cells. Thus, here we further studied the protein secretory pathway and specifically unraveled the mechanisms in the evolved strains that lead to improved α-amylase production by performing multi-omics analysis for two representative isolated strains, MH34 and B184, as well as for the original strain, AAC. MH34 and B184 were sequentially isolated from AAC (Fig. 1A), and whole-genome sequencing revealed that B184 inherited all 58 point mutations of MH34 and obtained 30 more point mutations (9). A previous study showed that the α-amylase yields of MH34 and B184 increased 1.21-fold and 4.04-fold, respectively, in batch culture compared to the yield of AAC (14). To avoid bias caused by differences in the growth rates of different strains and delays in protein synthesis, we used steady-state chemostat cultures at two dilution rates as the culture conditions in the present study. In addition, chemostat culture is also an important mode for recombinant protein production in an industrial setting (15). We performed absolute quantification of the proteome, transcriptome, and exo-metabolome for the three strains grown at two different dilution rates in chemostat cultures. Through systematic multi-omics analysis, we identified two different routes for improving protein production.

## RESULTS

### α-Amylase productivity in chemostat culture.

Previous work showed that a higher specific growth rate was coupled to higher recombinant protein production in chemostat cultures (16). In view of the maximum specific growth rates of these three strains (0.276/h, 0.329/h, and 0.310/h for AAC, MH34, and B184, respectively) (14), we cultured the strains at dilution rates of 0.1/h and 0.2/h. Compared with the reference strain AAC, we found that the α-amylase productivity of MH34 and B184 was significantly improved at both dilution rates (Fig. 1B), which is in agreement with the results obtained for batch cultures (14). However, in batch culture, B184 produced α-amylase with a 1.29-fold increase of yield and 47% increase of productivity compared with levels for MH34. In contrast, in chemostat cultures, we found that the yield or the productivity of B184 was only 14% greater than that of MH34 at 0.2/h and even 22% lower at 0.1/h, which indicated the different mechanisms for improved protein production between MH34 and B184 and the importance of specific growth rate on the regulation of the mechanisms.

### Cellular resource allocation revealed by quantitative multi-omics analysis.

We performed absolute quantitative proteome and transcriptome analysis for AAC, MH34, and B184 grown in steady-state chemostat cultures at dilution rates 0.1/h and 0.2/h, with measurement of 2,869 and 2,551 protein-transcript pairs, respectively. We found that the correlation between protein and mRNA abundances is improved at 0.2/h (*R^2^* of 0.50, 0.50, and 0.52 for AAC, MH34, and B184, respectively) compared to that at 0.1/h (*R^2^* of 0.37, 0.48, and 0.34, respectively) (see Fig. S1A to F in the supplemental material), which could suggest that at a higher specific growth rate, cells coordinate transcription and translation better to avoid redundancy or a shortage of transcripts, which helps to reduce energy expenditure. We also annotated mRNAs and proteins based on the Yeast Gene Ontology (GO)-Slim bioprocess mapper (17) and found that the proteome and transcriptome fractions allocated to a specific process are better correlated (*R^2^* of 0.66 to 0.78) (Fig. S1G to L) than individual proteins/transcripts.

10.1128/mBio.02743-20.1 FIG S1 Correlation between transcript and protein levels. (A to F) Correlation of absolute mRNA and protein abundances of AAC, MH34, and B184 in chemostat cultures at dilution rates of 0.1/h and 0.2/h. (G to L) Correlation of biological processes between the levels of transcript and protein under these six conditions. All transcripts and proteins were allocated to 99 Yeast GO-Slim biological processes. For each process, the ratio of allocated transcripts/proteins to total transcripts/proteins was used as the fraction value (mol/mol). Download FIG S1, TIF file, 0.2 MB. Copyright © 2020 Qi et al. 2020 Qi et al. This content is distributed under the terms of the Creative Commons Attribution 4.0 International license.

**FIG 2:**
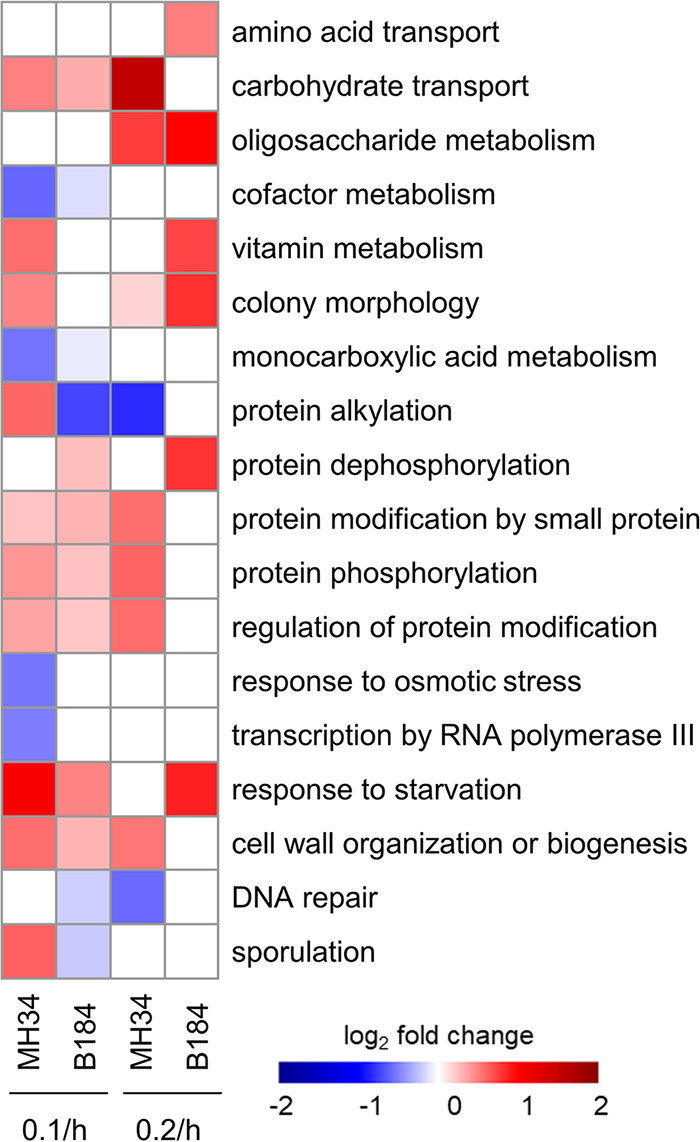
The differentially expressed biological processes (*P < *0.05) in MH34 and B184 revealed by proteome allocation. All proteins were allocated to the 99 Yeast GO-Slim biological processes. The proteome fraction (gram/gram) for each process was used. The expression levels of processes in AAC at 0.1/h and 0.2/h were used as the respective references.

Next, we analyzed the changes of specific bioprocesses in the proteome and transcriptome and listed the processes changing significantly (*P < *0.05) in MH34 or B184 as revealed by proteome (Fig. 2) and transcriptome (Fig. S2) analysis. Out of 99 Yeast GO-Slim bioprocesses, 78 were upregulated or downregulated at the transcriptome level, with a log_2_ fold change from −0.36 to 0.83. In the proteome, only 18 processes were differentially expressed, with the a log_2_ fold change from −0.82 to 1.55. Clearly, the transcriptome is remodeled in a global manner but at a smaller scale, while the proteome changes in a more specific manner that precisely impacts bioprocesses related to protein production, including amino acid transport, vitamin metabolism, protein modification, protein phosphorylation, and so on. The differences between transcriptome and proteome showed the reserves in translational capacity of yeast (18). Hence, we focused our analysis on differential expression at the proteome level between the three α-amylase production strains.

10.1128/mBio.02743-20.2 FIG S2 Overview of differentially expressed biological processes (*P < *0.05) in transcriptome. The expression levels of processes in AAC at 0.1/h and 0.2/h were used as the respective references. Download FIG S2, TIF file, 0.3 MB. Copyright © 2020 Qi et al. 2020 Qi et al. This content is distributed under the terms of the Creative Commons Attribution 4.0 International license.

### Significant factors relevant to protein production.

**FIG 3:**
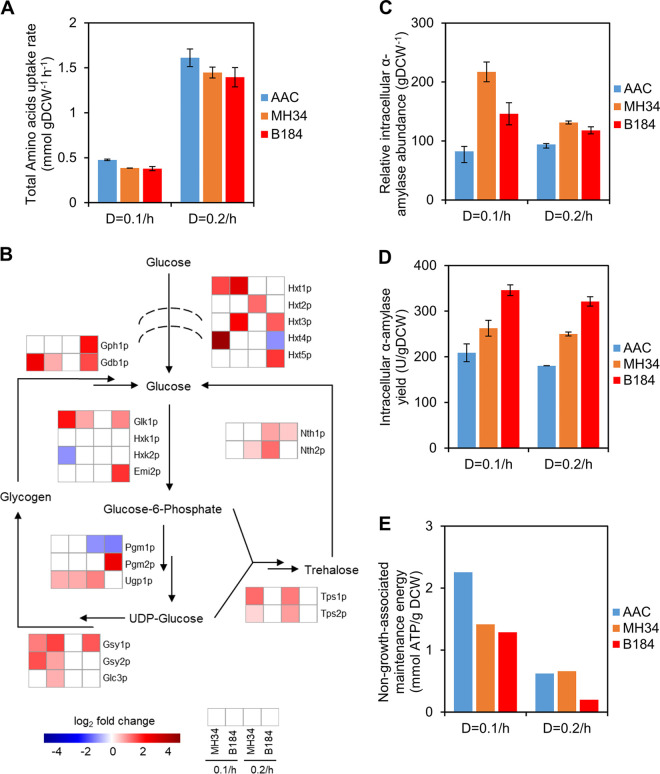
Overview of significant factors relevant to protein production. (A) Total amino acid uptake rates of strains in chemostat cultures. Data shown are mean values ± standard errors of the means of biological duplicates. (B) Differentially expressed proteins (*P < *0.05) related to glycogen and trehalose metabolism. The expression levels in AAC at 0.1/h and 0.2/h were used as the respective references. (C) Relative intracellular α-amylase abundance of strains in chemostat culture. Data shown are mean values ± standard errors of the means of biological duplicates. (D) Intracellular α-amylase yield of strains in chemostat culture. Data shown are mean values ± standard errors of the means of biological duplicates. (E) The non-growth-associated maintenance energy (NGAM) in chemostat cultures as calculated with help of the ecYeast8.3 model.

Since we observed differential expression of the process of amino acid transport at the proteome level (Fig. 2), we measured the amino acid uptake rates for 14 amino acids supplemented in our growth medium, which was optimized for protein production (19) (Fig. 3A). The total amino acid uptake rate for all strains was higher at 0.2/h than at 0.1/h, which means that it correlates well with the specific growth rate. However, compared with the rate for AAC, at both dilution rates the total uptake rate was low in MH34 and B184, which suggested that amino acid uptake may not be a limiting factor for α-amylase production. The higher amino acid uptake rate but lower productivity in AAC at 0.1/h could indicate less efficient metabolism in the strain, which was also revealed by previous work (14).

In addition to amino acid uptake, energy metabolism is also important for protein synthesis and secretion (20). We next investigated the abundance of proteins involved in carbohydrate transport and oligosaccharide metabolism, which were upregulated in the high-α-amylase production strains (Fig. 2). The upregulated proteins mainly participate in glycogen metabolism (Fig. 3B), which links carbohydrate metabolism to protein glycosylation and folding via the production of UDP-glucose (21). α-Amylase is a glycoprotein which carries one *N*-glycan, and UDP-glucose is a critical precursor of *N*-glycans, the changes in composition of which act as a signal that guides the folding process for glycoproteins. For example, when the *N*-glycan changes from Glc_3_Man_9_GlcNAc_2_ (G3M9) to Glc_1_Man_9_GlcNAc_2_ (G1M9), the protein is better folded by relevant enzymes and chaperones, and when it changes from Man_8_GlcNAc_2_ (M8) to Man_7_GlcNAc_2_ (M7), there is increased targeting of the protein to ERAD for degradation (3). Thus, the upregulation in glycogen metabolism is able to offer more available sugars, which could be recruited by *N*-glycans, and store more sugars that are trimmed from *N*-glycans, which could support the increased protein production in MH34 and B184.

Different from glycogen metabolism, trehalose metabolism showed differences between MH34 and B184. The proteins in trehalose metabolism were upregulated mainly in MH34 but not in B184 (Fig. 3B). Previous studies had shown that trehalose exerts bidirectional effects on protein folding (22, 23). On the one hand, trehalose can help to prevent folded proteins from denaturing and misfolded proteins from aggregating. On the other hand, trehalose interferes with refolding of denatured proteins by relevant molecular chaperones. Therefore, the upregulation of the trehalose cycle in MH34 suggests a dynamic control of the trehalose concentration in the cell, which could indicate that there are more misfolded proteins in MH34 than in B184.

To compare the fraction of misfolded α-amylase between the three strains, we measured the intracellular α-amylase abundance and activity (Fig. 3C and D). We found that in MH34 the abundance was greater but the activity was lower than that in B184, which means a higher fraction of misfolded α-amylase in MH34 than in B184.

We also investigated energy metabolism-related pathways, including glycolysis, the tricarboxylic acid (TCA) cycle, oxidative phosphorylation, and the pentose phosphate pathway (PPP), at the proteome level (Fig. S3). With the increase of specific growth rate from 0.1/h to 0.2/h, the abundance of glycolytic proteins in MH34 was increased while the expression levels of other pathways were maintained. In contrast, in AAC and B184 the expression of multiple enzymes involved in energy metabolism pathways was decreased. Specifically, the expression of proteins involved in glycolysis and the PPP decreased in AAC, while the expression of proteins involved in glycolysis, the TCA cycle, and the PPP decreased in B184. Since protein production needs a considerable fraction of energy in the overall cell metabolism (20), we assumed that the differences in energy metabolism also reflected the differences in protein synthesis and secretion.

10.1128/mBio.02743-20.3 FIG S3 Differential expression of energy metabolism-related pathways (*P < *0.05) in proteome at the dilution rate of 0.1/h to 0.2/h. The first row represents the change in abundances of total proteins working on energy metabolism-related pathways. Download FIG S3, TIF file, 0.1 MB. Copyright © 2020 Qi et al. 2020 Qi et al. This content is distributed under the terms of the Creative Commons Attribution 4.0 International license.

To test this hypothesis, we calculated the energy used for protein synthesis and secretion based on a genome-scale metabolic model with enzyme constraints (ecYeast8.3) (24). Energy used for protein production is a component of the non-growth-associated maintenance energy (NGAM) in ecYeast8.3, which we found to be always lower in B184 than in other strains (Fig. 3E). Especially compared with MH34, due to their similar α-amylase productivity levels in chemostat, the low NGAM level in B184 indicated that B184 expended less energy than MH34 to produce the same amount of correctly folded α-amylase, which was in line with the finding that the fraction of misfolded α-amylase was greater in MH34 than in B184.

### Regulation of *N*-glycans helps to increase the α-amylase yield.

The overall proteome allocation analysis revealed significant differences in protein synthesis-associated processes in addition to energy metabolism (Fig. 2 and 3). We next focused specifically on the protein secretory pathway, which involves more than 160 proteins that are responsible for different posttranslational processes in yeast. A previous yeast protein secretory model divided the secretory machinery into 16 subsystems (2). To reduce the complexity, we merged subsystems with similar functions and simplified the secretory pathway into 8 subsystems, including translocation, ER glycosylation, folding, ER glycosylphosphatidylinositol (GPI) anchoring, ERAD, trafficking between the ER and Golgi compartment, Golgi processing, and sorting. The components of each subsystem are listed in Data Set S1.

10.1128/mBio.02743-20.7 DATA SET S1 Components of protein secretory pathway in yeast. Download Data Set S1, XLSX file, 0.05 MB. Copyright © 2020 Qi et al. 2020 Qi et al. This content is distributed under the terms of the Creative Commons Attribution 4.0 International license.

**FIG 4:**
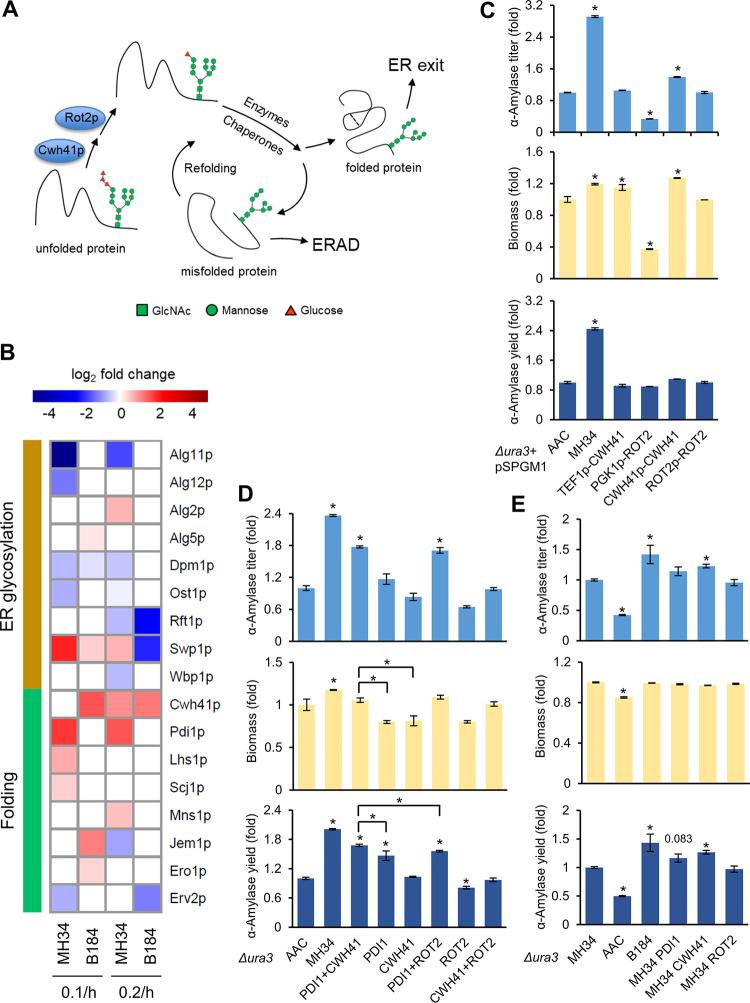
The protein folding pathway. (A) *N*-Glycan-directed protein folding pathway. (B) Differentially expressed proteins (*P < *0.05) related to the ER glycosylation process and the folding pathway. The expression levels in AAC at 0.1/h and 0.2/h were used as the respective references. (C) Promoter evaluation for overexpression of *CWH41* or *ROT2* from plasmids. The plasmid pSPGM1 was used for gene overexpression. (D) α-Amylase titer, biomass, and α-amylase yield of engineered strains in the background of strain AAC. (E) α-Amylase titer, biomass, and α-amylase yield of engineered strains in the background of MH34. For panels C, D, and E, data shown are mean values ± standard errors of the means of biological triplicates. Statistical significance was determined by a two-tailed Student's *t* test. *, *P < *0.05.

Proteome analysis revealed the differences in protein glycosylation and folding processes between strains. Since α-amylase is a kind of glycoprotein, the folding pathway is directed by the change of the composition of its *N*-glycans (Fig. 4A). Therefore, we further investigated the proteins involved to elucidate the specific contributors in these strains.

Within the ER glycosylation process, there were more downregulated proteins in MH34 than in B184 (Fig. 4B). Among them, Alg11p and Ost1p, which regulated the synthesis of *N*-glycan (3), are downregulated at both dilution rates in MH34 but not in B184. Correspondingly, within the folding process, the glucosidase Cwh41p, which catalyzes the first step in *N*-glycan trimming and initiates the folding process (Fig. 4A), was upregulated at both dilution rates in B184 and only at 0.2/h in MH34. On the other hand, Pdi1p was the only protein in the folding pathway to be upregulated at both dilution rates in MH34 (Fig. 4B). This is in line with its role in disulfide bond formation during protein folding as well as in guiding misfolded proteins to ERAD (3). Previous studies already reported that overexpression of *PDI1* can increase α-amylase production (10, 25).

To validate if the regulation of synthesis and trimming of *N*-glycans could increase protein yield and cause the differences between MH34 and B184, we studied the role of Cwh41p on protein production. First, we tested different promoters for overexpression. We overexpressed *CWH41* under the control of either a strong promoter or the *CWH41* native protomer carried by a multicopy-number plasmid in AAC. We also tested the overexpression of Rot2p, which catalyzes the second step in *N*-glycan trimming (Fig. 4A). The α-amylase titer increased with overexpression of *CWH41*, particularly when it was expressed under its native promoter, reaching a 40% increase in α-amylase production (Fig. 4C). Interestingly, the final biomass was significantly increased in this strain as well, which was rarely observed in previous engineering studies (10, 14, 26). In general, the final biomass is most often lower in strains engineered to overproduce recombinant proteins since increased protein production overloads the folding burden of the cell, leading to accumulation of misfolded proteins and increased energy expenditure in response to cell stress (27). One example is the overexpression of *PDI1*, which increased α-amylase yield but decreased final biomass (10). Here, the increases in both α-amylase production and final biomass suggest that *CWH41* overexpression improves protein production without the accumulation of misfolded proteins. In other words, *CWH41* overexpression might lead to more precise protein folding, consistent with the decreased fraction of misfolded α-amylase in B184.

A previous study reported that the overexpression of *PDI1* under the control of the *TEF1* promoter resulted in a higher α-amylase yield than overexpression using its native promoter (10). In the cases of *CWH41* and *ROT2*, the native promoters led to better results (Fig. 4C). Therefore, here we performed single-copy combinatorial overexpression in AAC with the superior promoter for each gene to further evaluate the role of Cwh41p (Fig. 4D). The data showed that the overexpression of *CWH41* itself did not increase the α-amylase yield in AAC, but the combinational overexpression of *PDI1* and *CWH41* did further increase the yield compared with that of overexpression of *PDI1* alone, which could indicate that the increase in folding precision is more important for strains with improved protein folding capacity. To further validate this, we engineered strain MH34, which has an improved folding capacity compared to that of AAC. The single-copy overexpression revealed that *CWH41* itself can help to increase the α-amylase yield by 27%, with a 43% increase in B184 under the same condition (Fig. 4E), which is in line with the observation that overexpression of *CWH41* helped to further increase the yield in an AAC strain already overexpressing *PDI1*. For *PDI1*, the expression level was already upregulated in MH34 (Fig. 4B), and overexpression therefore did not result in a significant difference. In addition to the single-copy integration, multicopy overexpression via plasmid in MH34 also showed that *CWH41* can increase the α-amylase yield (Fig. S4).

10.1128/mBio.02743-20.4 FIG S4 α-Amylase titer, biomass, and α-amylase yield of engineered strains in the background of MH34. The plasmid pSPGM1 was used for gene overexpression. Data shown are mean values ± standard errors of the means of biological triplicates. Statistical significance was determined by a two-tailed Student’s *t* test. *, *P < *0.05. Download FIG S4, TIF file, 0.05 MB. Copyright © 2020 Qi et al. 2020 Qi et al. This content is distributed under the terms of the Creative Commons Attribution 4.0 International license.

**FIG 5:**
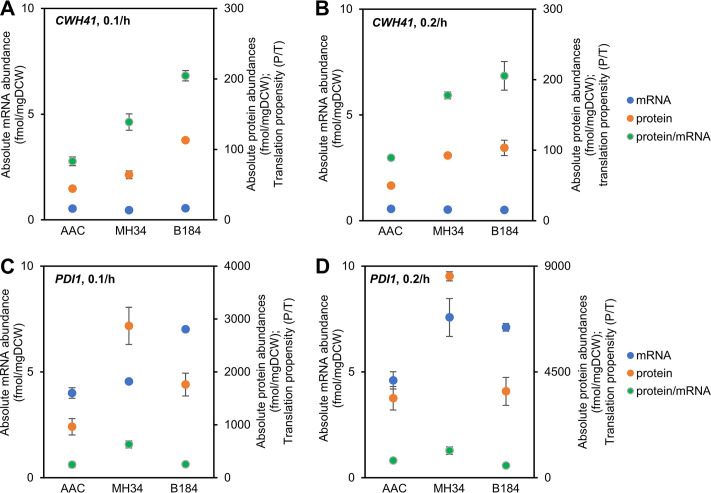
The mRNA abundance, protein abundance, and translation propensity (protein abundance/mRNA abundance) of *CWH41* or *PDI1* of strains in chemostat cultures.

To further study the regulatory mechanisms of Cwh41p and Pdi1p, the mRNA abundance, protein abundance, and translation propensity (protein abundance/mRNA abundance [protein abundance/mRNA abundance is abbreviated as P/T]) were determined (Fig. 5). For Cwh41p, compared with AAC, at the transcriptional level there was a limited increase in the evolved strains (less than 5% increase in B184 at 0.1/h and even a decrease under the other three conditions), and at the translational level the abundance significantly increased (over 100% increase in B184 at both dilution rates). Thus, the increase in Cwh41p mainly depends on posttranscriptional regulation. For Pdi1p, there was already a significant increase at the transcriptional level in the two evolved strains, but the different levels of posttranscriptional regulation led to different protein abundances (over 50% increase in the P/T value in MH34 at both dilutions and a decrease in the P/T value in B184). Thus, the regulation of Pdi1p needs both transcriptional and posttranscriptional regulation.

## DISCUSSION

In this study, we systematically analyzed the global differences among three S. cerevisiae strains to identify potential mechanisms for improved α-amylase production in the two evolved strains. In addition to the upregulated protein folding and protein modification processes, which help the evolved strains produce more α-amylase than the original strain, we also found significant differences between the two evolved strains at the proteome and protein production levels. More proteins working on the ER glycosylation process, such as Agl11p, Alg12p and Ost1p, were downregulated in MH34 than in B184 (27), and, correspondingly, MH34 produced a greater fraction of misfolded α-amylase and thus needed to allocate more energy for protein production at both dilution rates than B184. Further studies showed that the overexpression of *CWH41* can help MH34 increase α-amylase yield. Considering the role of Cwh41p in the protein folding process and the importance of protein glycosylation to the folding process of glycoproteins (28), we assumed that the overexpression of *CWH41* decreased the fraction of misfolded α-amylase in MH34 and thus increased the yield.

Examining the path from AAC to MH34 and then to B184 also reveals the precision of protein folding as a novel target for improving recombinant protein production through directed engineering. To date, efforts concerning the folding mechanisms to improve protein production have primarily targeted folding capacity and degradation of misfolded proteins (10, 29–31). Our study indicates that folding precision is another important component in this process, and its improvement can lead to higher recombinant protein yield.

Here, we identified that the glucosidase Cwh41p plays an important role in protein folding precision control, thus representing a valuable target that can be engineered for improving recombinant protein production. Unlike Pdi1p, for which abundance relies on both transcriptional and posttranscriptional regulation, Cwh41p mainly depends on posttranscriptional regulation, which is in line with the promoter selection for the overexpression of these two genes. The native promoter provided *CWH41* with better posttranslational regulation, and the strong promoter provided *PDI1* with better transcriptional regulation. The detailed regulatory mechanisms need further studies. In addition, many human diseases caused by protein misfolding (4), including Alzheimer’s disease, Parkinson’s disease, and type II diabetes, may benefit from further study of glucosidase I (GI) (3), which is the mammalian homologue of Cwh41p, and its control of protein folding precision as a possible target for new drugs to be used for treatment of these protein misfolding diseases.

## MATERIALS AND METHODS

### Strains, plasmids, and primers.

All strains, plasmids, and primers used in this study are listed in Table S1 in the supplemental material. Plasmids for gene overexpression were constructed by insertion of the gene fragment, which was amplified from the yeast genome by corresponding primer pairs and then assembled with the expression vector pSPGM1 through the Gibson assembly method. The guide RNA (gRNA) plasmid was constructed by recombining gRNA sequence-containing DNA fragments with vector backbone through the Gibson assembly method. The standard lithium acetate (LiAc)/single-stranded DNA (SS-DNA)/polyethylene glycol (PEG) method was used for yeast transformation.

10.1128/mBio.02743-20.5 TABLE S1 Strains, plasmids, and primers used in this study. Download Table S1, DOCX file, 0.03 MB. Copyright © 2020 Qi et al. 2020 Qi et al. This content is distributed under the terms of the Creative Commons Attribution 4.0 International license.

### Media and culture conditions.

For strain constructions, yeast strains were grown in synthetic dextrose-uracil (SD-URA) medium or yeast extract-peptone-dextrose (YPD) medium supplemented with 100 mg/liter nourseothricin at 30°C according to the auxotrophy or the resistance of the cells. For α-amylase production in shake flasks, yeast strains were cultured for 72 h at 200 rpm at 30°C with an initial optical density at 600 nm (OD_600_) of 0.1 in the SD-2×SCAA medium containing 20 g/liter glucose, 6.9 g/liter yeast nitrogen base without amino acids, 190 mg/liter Arg, 400 mg/liter Asp, 1,260 mg/liter Glu, 130 mg/liter Gly, 140 mg/liter His, 290 mg/liter Ile, 400 mg/liter Leu, 440 mg/liter Lys, 108 mg/liter Met, 200 mg/liter Phe, 220 mg/liter Thr, 40 mg/liter Trp, 52 mg/liter Tyr, 380 mg/liter Val, 1 g/liter bovine serum albumin (BSA), 5.4 g/liter Na_2_HPO_4_, and 8.56 g/liter NaH_2_PO_4_·H_2_O (pH 6.0), supplemented with 100 mg/liter uracil if needed. For bioreactor carbon-limited chemostat cultures, 20 g/liter glucose in the SD-2×SCAA medium was replaced by 7.5 g/liter glucose, and 5.4 g/liter Na_2_HPO_4_ and 8.56 g/liter NaH_2_PO_4_·H_2_O in the SD-2×SCAA medium were replaced by 2 g/liter KH_2_PO_4_ (pH 6.0). For MH34 and B184 at the dilution rate of 0.1/h, 2 ml of pure ethanol was added to the bioreactor to prevent oscillations. Overnight seed cultures which were grown to an OD_600_ of 3 to 4 were used to inoculate 400 ml of SD-2×SCAA medium in 1-liter bioreactor vessels (DasGip) with an initial OD_600_ of 0.01. The bioreactor system was run at 30°C with 600 rpm agitation and 24 liter/h airflow (pH 6; controlled by KOH).

### Sampling from bioreactor.

Samples for transcriptome, proteome, biomass, α-amylase, and exo-metabolome analysis were collected at the same time. First, the dead volume in the tube was collected and discarded. For transcriptome sampling, biomass was collected into chilled 50-ml Falcon tubes filled with 35 ml of crushed ice. Samples were centrifuged for 4 min at 3,000 × *g* at 4°C; cell pellets were washed once with 1 ml of chilled water, transferred into 1.5-ml Eppendorf tubes, and flash frozen in liquid nitrogen. For proteome sampling, biomass was collected into chilled 50-ml Falcon tubes. Samples were centrifuged for 4 min at 3,000 × *g* at 4°C; cell pellets were washed once with 20 ml of chilled water and once with 1 ml of chilled water, transferred into 1.5-ml Eppendorf tubes, and flash frozen in liquid nitrogen. All samples for transcriptome and proteome analysis were stored at −80°C until analysis. For biomass analysis, culture broth was collected into chilled 50-ml Falcon tubes. For α-amylase and exo-metabolome analysis, culture broth was collected into chilled 1.5-ml Eppendorf tubes. Samples were centrifuged for 10 min at 15,000 × *g* at 4°C; the supernatant was used for extracellular α-amylase and exo-metabolome analysis and stored at –20°C.

### RNA sequencing and quantification.

RNA was extracted using a Qiagen RNeasy minikit (Qiagen) according to the manufacturer’s protocol. RNA integrity was examined using a 2100 Bioanalyzer (Agilent Technologies). RNA concentration was determined using a Qubit RNA HS assay kit (Thermo Fisher). An Illumina TruSeq Stranded mRNA Library Prep kit (Illumina) was used to prepare mRNA samples for sequencing. Paired-end sequencing (2 by 150 bp) was performed on an Illumina NextSeq 500 system. Reads were quality controlled, mapped to the S. cerevisiae reference genome (Ensembl R64-1-1), and counted using the nf-core transcriptome sequencing (RNA-seq) pipeline (SciLifeLab, Stockholm, Sweden).

The absolute concentrations of 36 transcripts, covering the entire dynamic expression range, were measured using lysates of S. cerevisiae CEN.PK 113-7D cells. Linear regression between the absolute concentrations of these mRNAs and their corresponding fragments per kilobase per million (FPKM) values from RNA-seq were performed to obtain the slope and *y* intercept, which were used to quantify all mRNAs in this study. The *R^2^* value of the linear model was 0.92. The processed quantitative transcriptomics data are given in Data Set S2 in the supplemental material.

10.1128/mBio.02743-20.8 DATA SET S2 Absolute mRNA abundances in chemostat cultures. Download Data Set S2, XLSX file, 0.9 MB. Copyright © 2020 Qi et al. 2020 Qi et al. This content is distributed under the terms of the Creative Commons Attribution 4.0 International license.

### Quantitative proteome measurements.

All liquid chromatography-mass spectrometry (LC-MS) experiments were performed on an Orbitrap Fusion Tribrid mass spectrometer (Thermo Fisher) interfaced with an Easy-nLC1200 nanoflow liquid chromatography system (Thermo Fisher). Peptide and protein identification and quantification were performed using Proteome Discoverer, version 2.2 (Thermo Fisher), with Mascot (Matrix Science) as a database search engine.

The global relative protein quantification between the samples was performed via the modified filter-aided sample preparation (FASP) method, which included the two-stage digestion of each sample with trypsin in 1% sodium deoxycholate (SDC)–50 mM triethylammonium bicarbonate (TEAB) buffer and labeling with Tandem Mass Tag (TMT) 10-plex isobaric reagents (Thermo Fischer) according to the manufacturer’s instructions. The pooled reference sample was prepared from the aliquots of the lysates of S. cerevisiae CEN.PK 113-7D cells. The combined TMT-labeled set was prefractionated into 20 final fractions on an XBridge BEH C_18_ column (3.5-μm particle size; 3.0 by 150 mm; Waters Corporation) at pH 10, and each fraction was analyzed using a 60-min LC-MS method.

An intensity-based absolute quantification (iBAQ) approach was used to estimate the absolute protein concentrations in the pooled reference sample. An aliquot of 50 μg of the pooled sample was spiked with 10.6 μg of the UPS2 Proteomics Dynamic Range Standard (Sigma-Aldrich) and digested using the FASP protocol. The label-free data were processed using the Minora feature detection node in Proteome Discoverer, version 2.2, and the quantitative values from three technical (injection) replicates were averaged. Forty-three proteins from the UPS2 standard were detected with two or more unique peptides and used to calculate the linear regression coefficients between the known concentrations of the UPS2 proteins and their corresponding iBAQ measurements. The slope and *y* intercept of the linear regression were used to quantify the yeast proteins in the pooled reference sample. The absolute concentrations were calculated using the iBAQ-based absolute values for the pooled reference sample and the relative abundance values from the TMT experiment. The processed quantitative proteomics data are given in Data Set S3.

10.1128/mBio.02743-20.9 DATA SET S3 Absolute protein abundances in chemostat cultures. Download Data Set S3, XLSX file, 0.4 MB. Copyright © 2020 Qi et al. 2020 Qi et al. This content is distributed under the terms of the Creative Commons Attribution 4.0 International license.

### Biomass and exo-metabolome measurements.

For biomass measurements, 5 ml of culture broth was filtered by preweighed, 0.45-μm-pore-size filter paper. The cells were dried in a microwave at 360 W for 20 min and put in a desiccator for at least 3 days. For extracellular metabolite measurements, including those of glucose, ethanol, glycerol, pyruvate, succinate, and acetate, the quantification was performed using a high-performance liquid chromatography (HPLC) system (Thermo Fisher) with a Bio-Rad HPX-87H column (Bio-Rad) at the temperature of 45°C, and 5 mM H_2_SO_4_ was used as the elution buffer at a flow rate of 0.6 ml/min. For extracellular amino acid measurements, the quantification was performed through an LC-tandem MS (LC-MS/MS) system (Thermo Fisher) with an amino acid analyzer (AAA) C_18_ column (SCIEX). The method was developed according to the protocol of the aTRAQ reagents application kit (SCIEX).

### α-Amylase quantification.

The α-amylase activity was measured using an α-amylase assay kit (Megazyme) with a commercial α-amylase from Aspergillus oryzae (Sigma-Aldrich) as the standard. Samples were centrifuged for 10 min at 15,000 × *g* at 4°C, and the supernatant was used for extracellular α-amylase quantification. For intracellular α-amylase quantification, the cell pellet was washed with distilled water and resuspended in 500 μl of phosphate-buffered saline (PBS) with 5 μl of Halt protease inhibitor cocktail (Thermo Fisher). The cell suspension was added to a lysing matrix tube, and cell lysis was performed using a FastPrep-24 tissue and cell homogenizer (MP Biomedicals) by two 60-s cycles at a speed of 6.5 m/s (samples were put on ice for 5 min between the two cycles). Cell debris was removed by centrifugation, and the supernatant fraction was used for amylase quantification.

### Enzyme-constrained modeling.

The genome-scale metabolic model ecYeast8.3 (24) was used to generate enzyme-constrained models (ecModel) for each strain (AAC, MH34, and B184) at each dilution rate (0.1/h and 0.2/h) using the GECKO toolbox (32). Absolute quantitative proteome data were used as upper limits for the protein usages in the model. The default parameter 0.5 for the *f* factor was used in the generation of the models when the total protein pool was constrained. In order to measure the accessory energy that each strain generates except for biomass production, aerobic carbon-limited SD-2×SCAA medium was set in the model, and the non-growth-associated maintenance energy (NGAM) reaction was set as the objective function to represent the accessory energy. Exchange fluxes such as glucose uptake rate, amino acid uptake rates, and by-product production rates were set according to the measurements (Table S2). A value of 1.03 was set to be the flexible factor for fermentation exchange fluxes generated in the chemostat cultures in order not to overconstrain the model. Flux balance analysis (FBA) was used during the simulation.

10.1128/mBio.02743-20.6 TABLE S2 Exchange flux constraints used for ecModel construction. Download Table S2, DOCX file, 0.02 MB. Copyright © 2020 Qi et al. 2020 Qi et al. This content is distributed under the terms of the Creative Commons Attribution 4.0 International license.

### Data availability.

The RNA-seq raw data are available at Genome Expression Omnibus under accession number GSE147204 (33). The mass spectrometry proteomics data have been deposited with the ProteomeXchange Consortium via the PRIDE (34) partner repository under the identifiers PXD012803 (35) for the iBAQ data set and PXD018116 (36) for the TMT-based relative quantification data set. Reasonable requests for supporting data are available from the corresponding author.
