# Supplementary figures and images for: Different Routes of Protein Folding Contribute to Improved Protein Production in Saccharomyces cerevisiae
Source: mBio. 2020 Nov 10;11(6):e02743-20. doi: 10.1128/mBio.02743-20 (PMC7667031; doi:10.1128/mBio.02743-20)

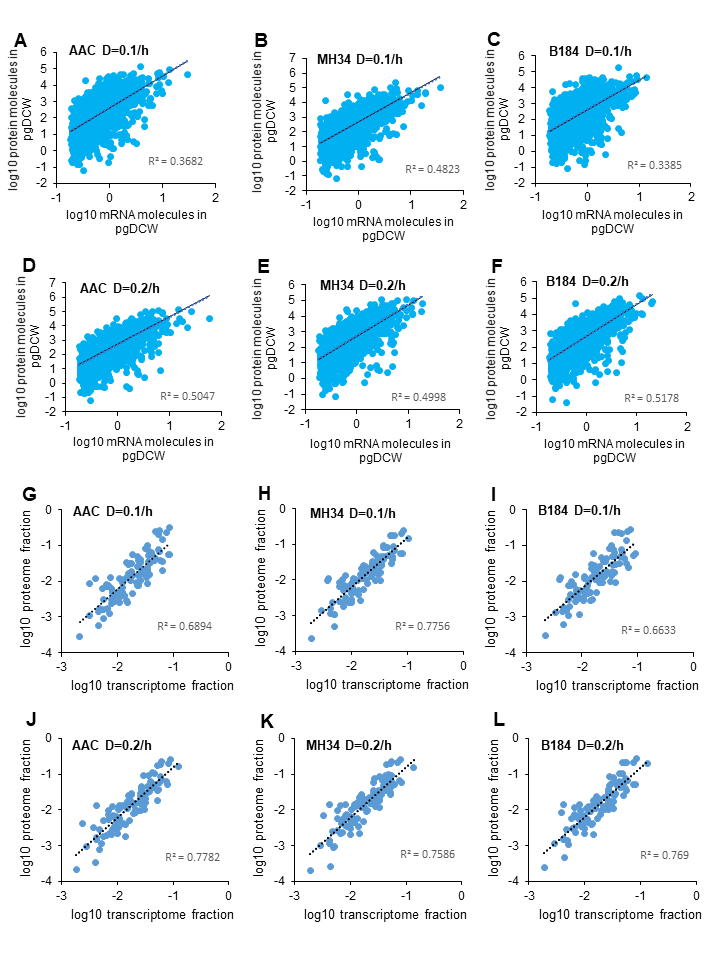

Supplement: FIG S1 [file mBio.02743-20-sf001.tif]

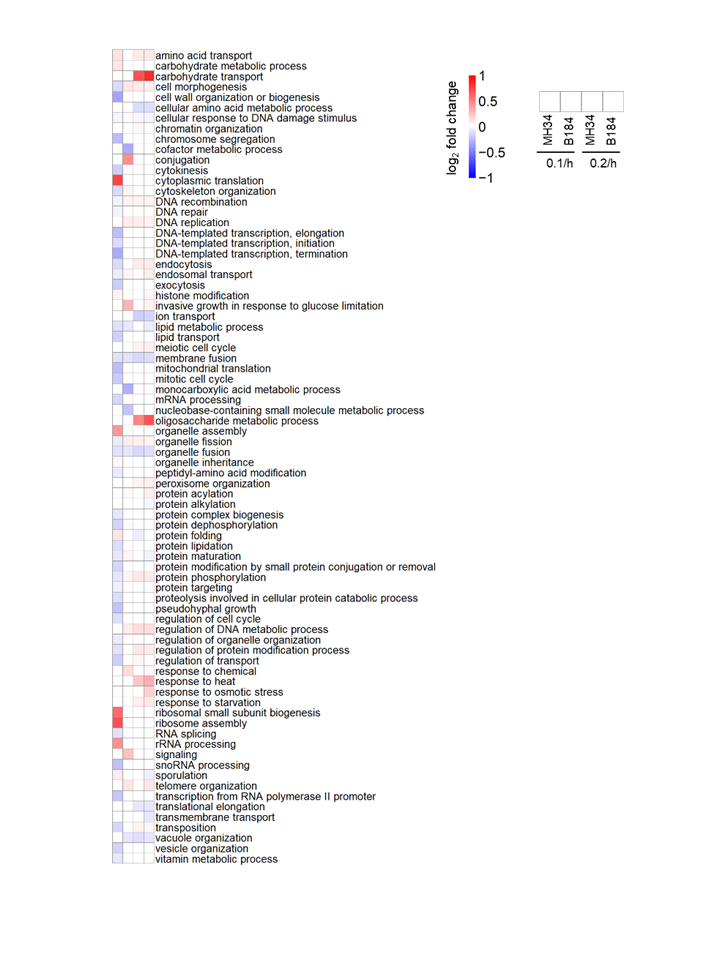

Supplement: FIG S2 [file mBio.02743-20-sf002.tif]

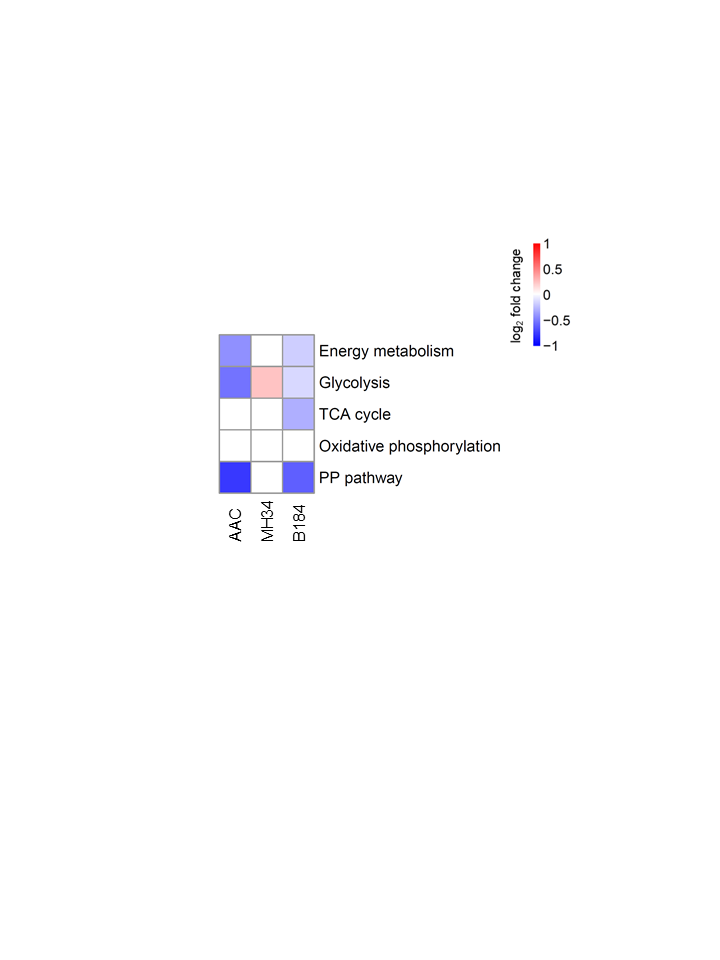

Supplement: FIG S3 [file mBio.02743-20-sf003.tif]

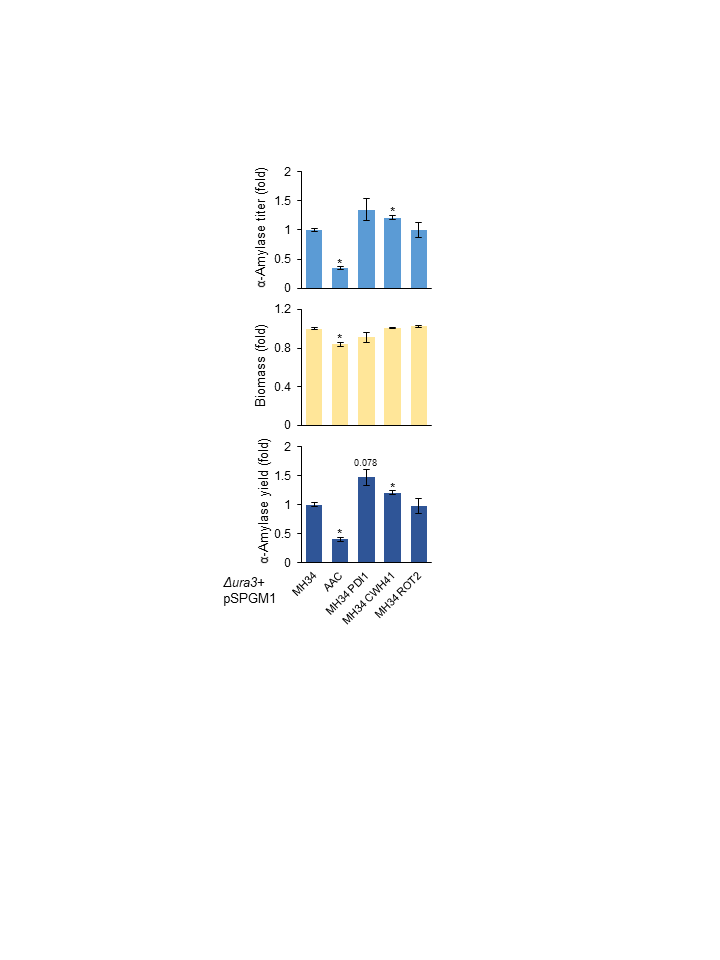

Supplement: FIG S4 [file mBio.02743-20-sf004.tif]
