# Supplementary material for: Different Routes of Protein Folding Contribute to Improved Protein Production in Saccharomyces cerevisiae
Source: mBio. 2020 Nov 10;11(6):e02743-20. doi: 10.1128/mBio.02743-20 (PMC7667031; doi:10.1128/mBio.02743-20)
Supplement: TABLE S1 [file mBio.02743-20-st001.docx]

Table S1. Strains, plasmids and primers used in this study

| **Strains and plasmids** | **Genotype** | **Reference** |
| --- | --- | --- |
| **Strains** |  |  |
| CEN.PK 530-1C | *MATa, URA3, HIS3, LEU2, TRP1, SUC2, MAL2-8^c^, tpi1(41-707)::loxP-KanMX4-loxP* | (1) |
| CEN.PK 530-1D | *MATa, ura3-52, HIS3, LEU2, TRP1, SUC2, MAL2-8^c^, tpi1(41-707)::loxP-KanMX4-loxP* | (2) |
| AAC | CEN.PK 530-1C + pAlphaAmyCPOT | (1) |
| MH34 | derived from AAC by UV mutagenesis | (3) |
| B184 | derived from MH34 by UV mutagenesis | (3) |
| Δura3 AAC (1D-AAC) | CEN.PK 530-1D + pAlphaAmyCPOT | (4) |
| Δura3+pSPGM1 AAC | 1D-AAC + pSPGM1 | This study |
| Δura3+pSPGM1 AAC TEF1p-CWH41 | 1D-AAC + pSPGM1-CWH41 | This study |
| Δura3+pSPGM1 AAC PGK1p-ROT2 | 1D-AAC + pSPGM1-ROT2 | This study |
| Δura3+pSPGM1 AAC CWH41p-CWH41 | 1D-AAC + pSPGM1-CWH41p-CWH41 | This study |
| Δura3+pSPGM1 AAC ROT2p-ROT2 | 1D-AAC + pSPGM1-ROT2p-ROT2 | This study |
| Δura3 AAC PDI1 | 1D-AAC + (TEF1p-PDI1-ADH1t) | This study |
| Δura3 AAC CWH41 | 1D-AAC + (CWH41p-CWH41-ADH1t) | This study |
| Δura3 AAC ROT2 | 1D-AAC + (ROT2p-ROT2-CYC1t) | This study |
| Δura3 AAC PDI1+CWH41 | 1D-AAC + (TEF1p-PDI1-ADH1t) + (CWH41p-CWH41-ADH1t) | This study |
| Δura3 AAC PDI1+ROT2 | 1D-AAC + (TEF1p-PDI1-ADH1t) + (ROT2p-ROT2-CYC1t) | This study |
| Δura3 AAC CWH41+ROT2 | 1D-AAC + (CWH41p-CWH41-ADH1t) + (ROT2p-ROT2-CYC1t) | This study |
| Δura3 MH34 (1D-MH34) | MH34 *ura3Δ::NatMX* | This study |
| Δura3 B184 (1D-B184) | B184 *ura3Δ::NatMX* | This study |
| Δura3 MH34 PDI1 | 1D-MH34 + (TEF1p-PDI1-ADH1t) | This study |
| Δura3 MH34 CWH41 | 1D-MH34 + (CWH41p-CWH41-ADH1t) | This study |
| Δura3 MH34 ROT2 | 1D-MH34 + (ROT2p-ROT2-CYC1t) | This study |
| Δura3+pSPGM1 MH34 | 1D-MH34 + pSPGM1 | This study |
| Δura3+pSPGM1 MH34 PDI1 | 1D-MH34 + pSPGM1-PDI1 | This study |
| Δura3+pSPGM1 MH34 CWH41 | 1D-MH34 + pSPGM1-CWH41p-CWH41 | This study |
| Δura3+pSPGM1 MH34 ROT2 | 1D-MH34 + pSPGM1-ROT2p-ROT2 | This study |
| **Plasmids** |  |  |
| pAlphaAmyCPOT | 2 μm, AmpR, *TPI1p-alpha factor leader-amylase gene-TPI1t, POT1* gene from *S. pombe* as a selection marker | (1) |
| pCfB3042 | 2 μm, AmpR, *TEF1p-NatMX-TEF1t* | (5) |
| pSPGM1 | 2 μm, AmpR, *URA3*, *TEF1p-ADH1t*, *PGK1p-CYC1t* | (6) |
| pSPGM1-PDI1 | 2 μm, AmpR, *URA3*, *TEF1p-PDI1-ADH1t*, *PGK1p-CYC1t* | (7) |
| pSPGM1-CWH41 | 2 μm, AmpR, *URA3*, *TEF1p-CWH41-ADH1t*, *PGK1p-CYC1t* | This study |
| pSPGM1-ROT2 | 2 μm, AmpR, *URA3*, *TEF1p-ADH1t*, *PGK1p-ROT2-CYC1t* | This study |
| pSPGM1-CWH41p-CWH41 | 2 μm, AmpR, *URA3*, *CWH41p-CWH41-ADH1t*, *PGK1p-CYC1t* | This study |
| pSPGM1-ROT2p-ROT2 | 2 μm, AmpR, *URA3*, *TEF1p-ADH1t*, *ROT2p-ROT2-CYC1t* | This study |
| pECAS9-gRNA-KlURA3-tX2 | 2 μm, AmpR, *KlURA3*, *TEF1p-eCas9-CYC1t*, *gRNA-X-2* | This study |
| **Primers** | **Sequence (5'-3')** | **Note** |
| **Plasmid construction** |  |  |
| SP-iCWH41-F | gcttgtaattaaaacttag | pSPGM1 backbone clone for *CWH41* insertion |
| SP-iCWH41-R | attaacaattcttcgccagagg | pSPGM1 backbone clone for *CWH41* insertion |
| SP-iROT2-F | agcttggtaccgcggctagctaa | pSPGM1 backbone clone for *ROT2* insertion |
| SP-iROT2-R | gatccttgttttatatttgttg | pSPGM1 backbone clone for *ROT2* insertion |
| CWH41-F | ctaatctaagttttaattacaagcatgcttatttcaaaatctaagatg | *CWH41* clone for insertion into pSPGM1 |
| CWH41-R | cctctggcgaagaattgttaattcagaagcgtccaaggatgt | *CWH41* clone for insertion into pSPGM1 |
| ROT2-F | ctttttacaacaaatataaaacaagatggtccttttgaaatggct | *ROT2* clone for insertion into pSPGM1 |
| ROT2-R | cttagctagccgcggtaccatcaaaaaataacttcccaatcttta | *ROT2* clone for insertion into pSPGM1 |
| SP-CWH41-1-F | aacctctgacacatgcagct | pSPGM1-CWH41 backbone 1 clone for *CWH41p* replacement |
| SP-CWH41-1-R | gcatgcttatttcaaaatct | pSPGM1-CWH41 backbone 1 clone for *CWH41p* replacement |
| SP-CWH41-2-F | ggccggcctggaagtaccttcaaa | pSPGM1-CWH41 backbone 2 clone for *CWH41p* replacement |
| SP-CWH41-2-R | agctgcatgtgtcagaggtt | pSPGM1-CWH41 backbone 2 clone for *CWH41p* replacement |
| SP-ROT2-1-F | gatggtccttttgaaatggc | pSPGM1-ROT2 backbone 1 clone for *ROT2p* replacement |
| SP-ROT2-1-R | gagacaataaccctgataaatgc | pSPGM1-ROT2 backbone 1 clone for *ROT2p* replacement |
| SP-ROT2-2-F | gcatttatcagggttattgtctc | pSPGM1-ROT2 backbone 2 clone for *ROT2p* replacement |
| SP-ROT2-2-R | aggccggccgcacacaccata | pSPGM1-ROT2 backbone 2 clone for *ROT2p* replacement |
| CWH41p-F | gaaggtacttccaggccggcccccgctttggaatcttctca | *CWH41p* clone for insertion into pSPGM1-CWH41 |
| CWH41p-R | agattttgaaataagcatgctgtgaagcaacctgaaccgt | *CWH41p* clone for insertion into pSPGM1-CWH41 |
| ROT2p-F | tatggtgtgtgcggccggcctggcgctctgggtcttttgac | *ROT2p* clone for insertion into pSPGM1-ROT2 |
| ROT2p-R | gccatttcaaaaggaccatcgttgcatacggttgattcga | *ROT2p* clone for insertion into pSPGM1-ROT2 |
| C9gRX2-1-F | ctctcgaagtggtcacgtgcgttttagagctagaaatagc | gRNA plasmid backbone 1 clone for replacement of gRNA targeting X2, the red sequence is the X2 gRNA sequence |
| C9gRX2-1-R | tatgagcttctcgaccgttc | gRNA plasmid backbone 1 clone for replacement of gRNA targeting X2 |
| C9gRX2-2-F | gaacggtcgagaagctcata | gRNA plasmid backbone 2 clone for replacement of gRNA targeting X2 |
| C9gRX2-2-R | gcacgtgaccacttcgagaggatcatttatctttcactgc | gRNA plasmid backbone 2 clone for replacement of gRNA targeting X2 |
| **Plasmid verification** |  |  |
| CWH41t-F | ccatagcttcaaaatgtttc | pSPGM1-CWH41 verification |
| CWH41t-R | cggatttttgaagtagtgac | pSPGM1-CWH41 verification |
| ROT2t-F | aagaaattaccgtcgctcgt | pSPGM1-ROT2 verification |
| ROT2t-R | gatattttcgaccagaatgag | pSPGM1-ROT2 verification |
| CWH41pt-F | aatatacgatcgggatac | pSPGM1-CWH41p-CWH41 verification |
| CWH41pt-R | tatacctgagaaagcaacct | pSPGM1-CWH41p-CWH41 verification |
| ROT2pt-F | cggtcaagtgataagtttc | pSPGM1-ROT2p-ROT2 verification |
| ROT2pt-R | ctggataaacgccggcgaaa | pSPGM1-ROT2p-ROT2 verification |
| C9gRt-F | cgggagtgtattgacgctgg | gRNA plasmid verification |
| C9gRt-R | ggactcctccagcctatgga | gRNA plasmid verification |
| ***URA3* deletion** |  |  |
| URA3up-F | ggttcatcatctcatggatc | *URA3* upstream fragment clone |
| URA3up-R-Nat | gtattctgggcctccatgtcgatttatcttcgtttcctgc | *URA3* upstream fragment clone |
| Nat-F | gacatggaggcccagaatac | *NatMX* fragment clone |
| Nat-R | cagtatagcgaccagcattc | *NatMX* fragment clone |
| URA3dn-F-Nat | gaatgctggtcgctatactgaaaactgtattataagtaaatgcatg | *URA3* downstream fragment clone |
| URA3dn-R | ttatcggcccaagccttgtc | *URA3* downstream fragment clone |

**Reference**

1. Liu Z, Tyo KEJ, Martínez JL, Petranovic D, Nielsen J. 2012. Different expression systems for production of recombinant proteins in *Saccharomyces cerevisiae*. Biotechnol Bioeng 109:1259–1268.

2. Hou J, Tyo K, Liu Z, Petranovic D, Nielsen J. 2012. Engineering of vesicle trafficking improves heterologous protein secretion in *Saccharomyces cerevisiae*. Metab Eng 14:120–127.

3. Huang M, Bai Y, Sjostrom SL, Hallström BM, Liu Z, Petranovic D, Uhlén M, Joensson HN, Andersson-Svahn H, Nielsen J. 2015. Microfluidic screening and whole-genome sequencing identifies mutations associated with improved protein secretion by yeast. Proc Natl Acad Sci 112:E4689–E4696.

4. Liu Z, Liu L, Österlund T, Hou J, Huang M, Fagerberg L, Petranovic D, Uhlén M, Nielsen J. 2014. Improved production of a heterologous amylase in *Saccharomyces cerevisiae* by inverse metabolic engineering. Appl Environ Microbiol 80:5542–5550.

5. Jessop-Fabre MM, Jakočiūnas T, Stovicek V, Dai Z, Jensen MK, Keasling JD, Borodina I. 2016. EasyClone-MarkerFree: A vector toolkit for marker-less integration of genes into *Saccharomyces cerevisiae* via CRISPR-Cas9. Biotechnol J 11:1110–1117.

6. Chen Y, Partow S, Scalcinati G, Siewers V, Nielsen J. 2012. Enhancing the copy number of episomal plasmids in *Saccharomyces cerevisiae* for improved protein production. FEMS Yeast Res 12:598–607.

7. Huang M, Wang G, Qin J, Petranovic D, Nielsen J. 2018. Engineering the protein secretory pathway of *Saccharomyces cerevisiae* enables improved protein production. Proc Natl Acad Sci 115:E11025–E11032.
