# Supplementary material for: Different Routes of Protein Folding Contribute to Improved Protein Production in Saccharomyces cerevisiae
Source: mBio. 2020 Nov 10;11(6):e02743-20. doi: 10.1128/mBio.02743-20 (PMC7667031; doi:10.1128/mBio.02743-20)
Supplement: TABLE S2 [file mBio.02743-20-st002.docx]

Table S2. Exchange flux constrains used for ecModel construction

|  | AAC-0.1/h | MH34-0.1/h | B184-0.1/h | AAC-0.2/h | MH34-0.2/h | B184-0.2/h |
| --- | --- | --- | --- | --- | --- | --- |
| P_tot_ (g gDCW^-1^) | 0.42 | 0.42 | 0.42 | 0.42 | 0.42 | 0.42 |
| glucose uptake (mmol gDCW^-1^ h^-1^) | 2.026949029 | 1.567936407 | 1.537793561 | 2.569971571 | 3.208706 | 2.61378646 |
| Arg uptake (mmol gDCW^-1^ h^-1^) | 0.052343038 | 0.053079771 | 0.052673734 | 0.053932727 | 0.054185763 | 0.04667075 |
| Asp uptake (mmol gDCW^-1^ h^-1^) | 0.215556263 | 0.151844049 | 0.142247009 | 0.170878547 | 0.173481956 | 0.141838005 |
| Glu uptake (mmol gDCW^-1^ h^-1^) | 0.53159071 | 0.296546135 | 0.307981129 | 0.44867443 | 0.458574084 | 0.360717284 |
| Gly uptake (mmol gDCW^-1^ h^-1^) | 0.091180537 | 0.107246827 | 0.099962638 | 0.066441886 | 0.072313861 | 0.066604609 |
| His uptake (mmol gDCW^-1^ h^-1^) | 0.060901338 | 0.054173666 | 0.048421784 | 0.047142728 | 0.047659799 | 0.04031022 |
| Ile uptake (mmol gDCW^-1^ h^-1^) | 0.143539736 | 0.125005615 | 0.109922297 | 0.112610597 | 0.114392288 | 0.098159191 |
| Leu uptake (mmol gDCW^-1^ h^-1^) | 0.234080876 | 0.196221686 | 0.169380337 | 0.16459354 | 0.170575427 | 0.147746313 |
| Lys uptake (mmol gDCW^-1^ h^-1^) | 0.175935643 | 0.142013039 | 0.130724579 | 0.159781776 | 0.161949524 | 0.136669893 |
| Met uptake (mmol gDCW^-1^ h^-1^) | 0.071346734 | 0.055422881 | 0.053599179 | 0.047015084 | 0.044934446 | 0.040509703 |
| Phe uptake (mmol gDCW^-1^ h^-1^) | 0.096209991 | 0.079928138 | 0.072517436 | 0.065415978 | 0.06700728 | 0.058747248 |
| Thr uptake (mmol gDCW^-1^ h^-1^) | 0.157136295 | 0.127500391 | 0.116028484 | 0.116421817 | 0.112609234 | 0.09803052 |
| Trp uptake (mmol gDCW^-1^ h^-1^) | 0.017863343 | 0.014271675 | 0.013834162 | 0.012937414 | 0.012978026 | 0.011695828 |
| Tyr uptake (mmol gDCW^-1^ h^-1^) | 0.024977497 | 0.020612303 | 0.019492225 | 0.01692814 | 0.017257843 | 0.01551929 |
| Val uptake (mmol gDCW^-1^ h^-1^) | 0.195541751 | 0.171038156 | 0.148823372 | 0.157448998 | 0.160021851 | 0.134379031 |
| CO_2_ production (mmol gDCW^-1^ h^-1^) | 6.259201412 | 4.842124095 | 5.731696905 | 6.016296311 | 6.139778424 | 5.624092759 |
| pyruvate production (mmol gDCW^-1^ h^-1^) | 0.003003499 | 0.003116136 | 0.002010884 | 0.008798837 | 0.013527388 | 0.009043104 |
| succinate production (mmol gDCW^-1^ h^-1^) | 0 | 0 | 0 | 0 | 0 | 0 |
| glycerol production (mmol gDCW^-1^ h^-1^) | 0.03857589 | 0.172304186 | 0.07510447 | 0.500915714 | 0.378783129 | 0.256741346 |
| acetate production (mmol gDCW^-1^ h^-1^) | 0 | 0.196093813 | 0.251298718 | 0.428893556 | 0.29318302 | 0.311421819 |
| ethanol production (mmol gDCW^-1^ h^-1^) | 0 | 0 | 0 | 0.202464707 | 1.760815902 | 0.846638824 |
